# Moral dilemmas in virtual reality: a narrative review of findings and methodological challenges

**DOI:** 10.3389/fpsyg.2025.1701188

**Published:** 2025-12-08

**Authors:** Margherita Pucci, Lorenzo Gagliardi

**Affiliations:** 1 Department of Neuroscience, University of Padua, Padua, Italy; 2 Department of Developmental Psychology and Socialization, University of Padua, Padua, Italy

**Keywords:** moral decision-making, moral dilemmas, virtual reality, human-computer interaction, self-driving cars

## Abstract

Moral dilemmas have long been challenging to study, as different methods often produce results that are hard to compare depending on how questions are framed and responses elicited. Over the past two decades, however, Virtual Reality (VR) has revolutionized this field by the means of immersive and ecologically valid simulations that better reflect real-life scenarios. This literature review examines the use of VR technologies to investigate moral dilemmas, focusing on how immersive environments affect moral decision-making. By analyzing 29 empirical and theoretical studies, we explore how VR enhances ecological validity, bridges the gap between moral judgment and action, and allows researchers to simulate ethically challenging scenarios that would be impossible to recreate in real life. The review covers classic dilemmas—such as the trolley and footbridge problems—as well as applied contexts like driving scenarios, autonomous vehicle decision-making, and professional ethics training. Across these settings, VR studies consistently reveal a general trend toward utilitarian choices, modulated by contextual factors (e.g., age of victims, time pressure), individual differences, and emotional salience. Furthermore, the paper highlights VR’s capacity to integrate multimodal data, such as physiological responses and eye-tracking, providing a richer picture of moral cognition. In a dedicated section, we review the main methodological arguments in favor of using VR, along with critiques concerning ecological validity, replicability, and ethical risks linked to virtually real experiences. We conclude by outlining recommendations for future research, emphasizing the importance of expanding VR applications, improving methodological standardization, and developing ethically responsible immersive paradigms.

## 1 Introduction

The verb “to decide” derives from the Latin *decīdĕre*, a compound of *de-* and *caedĕre* (“to cut”), literally meaning “to cut off.” It refers to the act of putting an end to a problematic situation by making a judgment and/or choosing between different options. As Jean-Paul Sartre famously said, “we cannot not choose”: every day of our lives we are called upon to make choices, ranging from the simplest to the most complex. Every action we take results from a decision made with varying degrees of awareness and ease.

A moral dilemma is a situation in which a person is required to make a difficult decision by appealing to their own principles and values to determine what is right and wrong—or at least which among the available options is the most acceptable for themselves, their conscience, and others. When faced with a moral decision, the individual experiences an internal conflict that must be resolved, as the choice made will lead to outcomes that may be both defensible and controversial. Over the years, an increasing number of scholars—including psychologists, neuroscientists, and philosophers—have become interested in understanding the cognitive and moral processes involved in decision-making under conditions of high emotional and ethical pressure.

As Wilson (1975) first suggested in his call to “biologicize morality,” the foundations of moral judgment lie in a limited set of affective responses shaped by natural selection and rooted in the emotive centers of the brain. This biological perspective inspired later models emphasizing the intuitive nature of moral evaluation. In particular, Haidt’s (2001) Social Intuitionist Model (SIM) posits that moral judgments arise primarily from rapid, automatic emotional intuitions, while deliberate reasoning often serves a *post hoc* justificatory function. Complementing this view, Greene et al. (2001, 2004) provided neuroscientific evidence for a dual-process model of moral decision-making, suggesting that deontological judgments are driven by automatic emotional processes, whereas utilitarian decisions rely on controlled, cognitive reasoning. Together, these perspectives converge in depicting morality as a dynamic interplay between emotion and reason, intuition and deliberation.

Building on this growing interest, this work critically evaluates the emerging field that uses immersive technologies—particularly virtual reality—to investigate moral dilemmas and the processes underlying them. One of the earliest and most well-known moral dilemmas used to investigate individuals’ moral decision-making is the so-called *trolley problem* (Foot, 1967; Thomson, 1985), more recently reported by Patil et al. (2014) as follows:

“A runaway trolley is headed for five people who will be killed if it proceeds on its present course. The only way to save them is to hit a switch that will turn the trolley onto an alternate set of tracks where it will kill one person instead of five. Is it appropriate for you to turn the trolley in order to save five people at the expense of one?”

Faced with this dilemma, several studies—including those by Greene et al. (2001, 2004)—have shown that the majority of individuals judge it morally acceptable to pull the lever, redirecting the trolley and thereby killing one person in order to save five. This is considered a utilitarian response that aims to minimize overall harm by choosing the “lesser evil.”

However, in a slightly modified version of the trolley problem—known as the *footbridge dilemma* (Thomson, 1985)—people’s responses change dramatically. In this case, the dilemma reads as follows:

“A runaway trolley is heading toward five people who are on the tracks. You are standing on a footbridge above the tracks, next to a large man. The only way to save the five people is to push this man off the bridge so that his body will stop the trolley. He will die, but the five people will be saved. Is it morally permissible to push the man off the bridge to save five people?”

Despite the structural similarity to the trolley dilemma—sacrificing one person to save five—most individuals respond that they would not push the man. This is typically interpreted as a *deontological* judgment, grounded in the principle that killing an innocent person is inherently wrong, regardless of the consequences.

According to Greene et al. (2004), the divergent decisions observed in these scenarios can be explained by the very nature of the dilemmas themselves, which fall into two distinct categories he defines as *personal* and *impersonal*.^1^ The classic trolley dilemma belongs to the category of *impersonal moral dilemmas*, in which individuals are able to make decisions in a more rational manner and tend to feel less personally responsible for the consequences of their actions. In contrast, *personal dilemmas*—such as the footbridge dilemma—require the individual to actively push another person off a bridge to achieve the same outcome as in the trolley dilemma, that is, saving five lives at the cost of one. However, in this case, the physical and emotional involvement in the action makes the individual feel more directly responsible for the harm inflicted, thus increasing the likelihood of judging the action as morally unacceptable.

The study of moral dilemmas is therefore far from straightforward, and the methods used to investigate them have been numerous and often difficult to compare, partly due to the nature of the questions asked, which could elicit different types of responses. Over the last two decades, however, a new technological paradigm has transformed research in this field. Specifically, the introduction of Virtual Reality (hereafter, VR) has significantly changed how moral dilemmas are studied, enhancing the ecological validity of experiments and bringing them closer to real-world moral decision-making.

It is useful in this context to provide some basic definitions of these technologies to illustrate the inclusion criteria we adopted for this review. Previous VR studies on moral dilemmas partially neglected to precisely define VR, probably due to the increasing notoriety of such instruments even beyond scientific research. However, other fields such as health-care (for a review, see Kardong-Edgren et al., 2019) and social neuroscience (Parsons et al., 2017) have produced overall convergent definitions of virtual reality, such as “simulations that use a variety of immersive, highly visual, 3D characteristics to replicate real-life situations” (Lopreiato et al., 2016) or “computer-generated environment in which the user can perceive, feel and interact in a manner that is similar to a physical place” (Parsons et al., 2017, p. 2). A key feature of these technologies is that, compared to regular computer-screen simulations, VR involves three dimensional environments that gives an “immersion effect” (Lopreiato et al., 2016, p. 40). Such immersion is described as the psychological reaction to virtual stimuli, “which causes the participant to be enveloped by and interact with an environment that provides a continuous stream of virtual and haptic stimuli and experiences” (Kardong-Edgren et al., 2019, p. 29). Ideally, in a fully effective virtual reality experience, “the user would not be able to distinguish an artificial environment from its physical counterpart” (Parsons et al., 2017, p. 2). The higher the level of immersion, the higher the sense of presence, which in turn is defined as the medium-induced sense of physically being in a given place (Witmer and Singer, 1998).

VR allows researchers to simulate complex scenarios in a controlled environment, overcoming one of the main criticisms directed at previous studies—that they primarily assessed moral *judgments*, which do not always align with the *actions* individuals might take in real life (e.g., Patil et al., 2014). As such, VR offers a promising solution to this limitation, making research in this area more informative and better suited to understanding the kinds of moral choices people make in various real-world contexts, from ethical decision-making in healthcare settings to the moral programming of autonomous vehicles.

Thus, the aim of this paper is two-fold. First, we aim to review empirical results coming from research on moral dilemmas conducted by the means of virtual environments. This review can be classified as a literature review in Grant and Booth’s (2009) framework. Second, we aim to summarize methodological discussions related to the use of virtual environments, reviewing the reasons to adopt these new technological tools and their limitations, as reported by authors of the papers we review. This work may provide useful insights on the state of the art in the field of moral psychology and its applications to different contexts. Furthermore, this study may provide guidance for future research employing virtual environments, as it highlights critical limitations of this emerging methodology.

To our knowledge, this is the first paper to directly review empirical results of VR studies in the field of moral dilemmas. In fact, although previous works have already covered more general topics such as the psychology of moral dilemmas (e.g., Cushman and Young, 2009), neuro-scientific dimensions (e.g., Blair et al., 2006; Christensen and Gomila, 2012) and psychometric properties (e.g., Ni et al., 2023), no previous work has specifically focused on reviewing VR applications to the study of moral dilemmas.

Section 2 explains the methodology we adopt for this review. Section 3 includes results, discussing VR studies on moral dilemmas divided by context of application (classic and applied moral dilemmas) and methodological implications. Finally, Section 4 discusses results, highlighting strengths and weaknesses, and introducing possible new lines of research.

## 2 Methods

For the purpose of this study, we will focus on studies using VR technologies, as defined in the previous section of the manuscript.

The bibliographic search was conducted using Google Scholar, PubMed and Web of Science because of their wide accessibility and inclusion of interdisciplinary sources.

Specifically, to ensure a comprehensive literature search, we carefully selected keywords that captured both the technological and the psychological dimensions of our topic. Specifically, we combined terms related to the technological component (“virtual reality,” “virtual environments,” “immersive technology”) with those describing the moral and cognitive aspects (“moral dilemmas,” “moral decision-making,” “moral cognition,” “ethics”). The selection of these keywords was based on their relevance to the intersection between moral psychology and immersive technologies. To ensure adequate coverage across different applied fields, we also included terms referring to specific application domains, such as “self-driving cars,” “helping professions,” and “human-computer interaction.” Boolean operator (“AND”) was applied to generate comprehensive combinations, allowing us to identify studies that examined the use of VR in both classic moral paradigms (e.g., trolley-type dilemmas) and applied contexts (e.g., autonomous vehicles, healthcare, and professional ethics).

Studies were included if they employed VR as part of the experimental design, and investigated moral or ethical decision-making processes. Exclusion criteria included pre-prints and dissertations. Regarding the typology of papers included in this review, we focused on experimental contributions for the sections dedicated to reviewing studies on moral dilemmas using VR, but also theoretical contributions and commentaries for the section dedicated to the methodological discussion about using VR in this branch of literature. The final set of papers include a total of 29 papers, of these 26 are empirical contributions and 3 methodological or conceptual studies.

## 3 Results

Research on moral dilemmas using VR has primarily aimed to replicate classic findings from text-based paradigms—such as the trolley and footbridge dilemmas—while also expanding into more applied, real-world contexts, including self-driving vehicles and helping professions. Accordingly, this section first presents empirical findings, organized by application domain (i.e., classic vs. applied moral dilemmas), and then examines key methodological reflections offered by previous authors to justify or criticize the adoption of VR in this area of research. For a summary of results, see Table 1 (for classic moral dilemmas) and Table 2 (for applied moral dilemmas).

**Table 1:** Summary of virtual reality studies investigating classic moral dilemmas.

Authors (year)	Sample / design	Dilemma	Key manipulations	Main findings
Pan and Slater (2011)	*N* = 116; Between-subjects	Modified trolley	Immersive VR vs. Desktop VR	89% utilitarian choices in immersive VR vs. 67% in desktop VR (in action condition).
Navarrete et al. (2012)	*N* = 293; Between-subjects	Trolley	Action vs. omission	90% utilitarian in action vs. 88% in omission; higher arousal in action; arousal associated with decreased utilitarianism.
Patil et al. (2014)	*N* = 40; Within-subjects	Trolley	VR vs. text-based	95% utilitarian in VR vs. 76% in text-based; arousal associated with increased utilitarianism.
Skulmowski et al. (2014)	*N* = 66; Mixed	Trolley	Number of victims; avatar properties; orientation in space;	96% utilitarian in 10 to 1 condition; males sacrificed more; no significant result for body orientation; pupil dilation increased after decision.
Francis et al. (2016)	*N* = 40 (Study 1) *N* = 62 (Study 2); Mixed	Footbridge	Action (= VR) vs. judgment (= survey) (in both studies)	Study 1: 70% utilitarian in Action vs. 20% in judgment; Study 2: 63.3% utilitarian in Action vs. 10% in judgment; higher heart rate associated with VR.
Francis et al. (2017)	*N* = 40 (Study 1) *N* = 25 (Study 2); Mixed	Footbridge	Action (= VR) vs. judgment (= survey); Haptic feedback	Study 1: 54% utilitarian in Action vs. 41% in judgment; Study 2: 56% utilitarian in Haptic Action condition vs. 63.3% in the regular VR Action condition;
Francis et al. (2019)	*N* = 48; Mixed	Footbridge	High Alcohol vs. Low alcohol vs. Placebo; Action (= VR) vs. judgment (= survey)	High alcohol: 68.75% utilitarian in Action vs. 6.25% in judgment; Low alcohol: 68.75% utilitarian in Action vs. 6.25% in judgment; Placebo: 75% utilitarian in Action vs. 25% in judgment; No main effect of condition and no interaction; higher heart rate associated with VR
Niforatos et al. (2020)	*N* = 60; Between-subjects	Mad bomber, Trolley	VR vs. text-based	Mad Bomber: no significant differences in decision to yield, use water-boarding, imprison or causing death; higher physical tactics used in VR, and higher psychological tactics used in text-based; Trolley: 82% utilitarian in VR vs. 76.7% in text-based
Terbeck et al. (2021)	*N* = 107; Between-subjects	Footbridge	Immersive VR vs. text-based	43% utilitarian in VR vs. 30.8% in text-based; in VR, participants gave more other-focused justifications for their choice; in text-based, participants gave more self-focused justifications.
Richesin et al. (2022)	*N* = 119; Between-subjects	Trolley	Prior contextual information on the dilemma vs. no information	85% utilitarian in informed group vs. 78% in uninformed group (not significant)
Maćkiewicz et al. (2023)	*N* = 82 (Study 1); Mixed *N* = 36 (Study 2); Within-subjects	Footbridge	Study 1: Action (=VR) vs. judgment (= survey);pushing vs. gesture Study 2: only VR; pushing vs. gesture	Study 1: Action: 52.63% utilitarian in pushing vs. 55.26% utilitarian in gesture; Judgment: 18.18% utilitarian in pushing vs. 27.27% utilitarian in gesture; Study 2: 63.89% utilitarian in pushing vs. 63.89% in gesture
Kissel et al. (2023)	*N* = 33; Between-subjects	Trolley and Footbridge (using in-home VR)	Switch (impersonal) vs. push (personal)	100% utilitarian in switch vs. 60% in push
Liu et al. (2025)	*N* = 60 (Study 1) *N* = 60 (Study 2); Mixed both studies	Trolley and Footbridge	Study 1: High vs. low cognitive load; VR vs. text-based; personal vs. impersonal Study 2: High vs. low cognitive load; pain images vs. non-pain images (empathy); personal vs. impersonal	Study 1 found significant differences across all conditions: 20% utilitarian in high load vs. 33% in low load; 36% utilitarian in VR vs. 18% in text-based; 36% utilitarian in impersonal dilemma vs. 17% in personal; Study 2 found significant differences across all conditions: 40% utilitarian in the empathy for pain group vs. 20% in the empathy for non-pain group; 38% utilitarian in impersonal dilemma vs. 22% in personal dilemma

**Table 2:** Summary of virtual reality studies investigating applied moral dilemmas, including driving scenarios, autonomous vehicles, and helping professions.

Authors (year)	Sample/design	Context	Key manipulations	Main findings
Frison et al. (2016)	*N* = 40; Within-subjects	Self-driving cars	Pedestrian features (age, number, personality); Driver’s survival probability	Driver’s probability of survival increases likelihood of saving others’ lives; Self-sacrifice (82,2%) more common if children are involved
Bergmann et al. (2018)	*N* = 189; Within-subjects	Driving decisions	Pedestrian features (number, age, likelihood of injury, innocence)	Utilitarian pattern in the VR-simulated driving trolley problem; high willingness to self-sacrifice (70%) when number of pedestrians is 5–7; children are saved more often than adults (90%) and elderly (91%)
Francis et al. (2018)	*N* = 60; Mixed	Helping professions training through footbridge dilemma	Professionals vs. lay; Action (= VR) vs. judgment (= survey)	Utilitarian choices more common in VR vs. text; no significant difference between groups in action or judgment; professionals show less arousal and regret compared to lay participants
Sütfeld et al. (2017)	*N* = 105; Between-subjects (decision time)	Driving decisions	Obstacle type (inanimate objects, animals, humans); decision time (slow vs. fast);	Consistent, utilitarian behavior, preferring to minimize total harm (humans over animals/objects, young over old). Simple value-of-life-based models accurately predicted decisions. Under time pressure, decision consistency decreased.
Faulhaber et al. (2019)	*N* = 189; Within-subjects	Driving decisions	Pedestrian features (number, age); contextual factors (street vs. sidewalk)	Consistent, utilitarian behavior, preferring to minimize total harm, with saving younger over old. Context had limited effect. Willingness to self-sacrifice more common when ≥5 others could be saved. Results support a “quantitative greater good” model and suggest a potential utilitarian ethics setting for autonomous vehicles.
Sütfeld et al. (2019)	*N* = 85 (Study 1) *N* = 93 (Study 2); Mixed	Driving decisions	Level of abstraction (naturalistic 3D environment vs. text-based), presentation modality (VR vs. 2D desktop); decision time (slow vs. fast); pedestrian features (age, gender)	Consistent, utilitarian behavior, preferring to minimize total harm, with saving younger over old and female over male. Time pressure reduced utilitarianism. Presentation modality and abstraction had minimal effect.
Samuel et al. (2020)	*N* = 32; Between-subjects	Driving decisions	Number of pedestrians (1 vs5 5; 0 vs. 5)	All the drivers made decisions under time pressure (2 s) and low visibility, failing to make utilitarian decisions (1 vs. 5: 43%; 0 vs. 5: 62.5%). Eye-tracking shows functional fixation (fewer glances to left/right in critical interval).
Benvegnù et al. (2021)	*N* = 15; Within-subjects	Self-driving cars	Role (driver vs. passenger of a self-driving car); Pedestrian features (age, number);	Utilitarian behavior, preferring to minimize total harm, with saving younger over old. No self-sacrifice was observed. Higher arousal, more negative valence, and greater perceived responsibility when driving themselves vs. when in a self-driving car.
De Melo et al. (2021)	*N* = 94 (Study 1), *N* = 276 (Study 2), *N* = 111 (Study 3), *N* = 200 (Study 4); Within-subjects	Self-driving cars	Risk of injury to driver and pedestrians; social influence (others’ utilitarian vs. non-utilitarian choices)	Utilitarian behavior, preferring to minimize total harm, but decreasing with higher risk to driver and increasing with lower risk to pedestrians. Participants became more utilitarian when others did the same.
Wang et al. (2023)	*N* = 60; Mixed	Driving decisions	Pedestrian features (age, gender); time pressure	Utilitarian behavior, largely preferring to self-sacrifice to minimize total harm, especially to save children or females. Higher self-sacrifice among female participants. Time pressure increased self-sacrifice.
Zhou and Zhu (2023)	*N* = 87 (Study 1) *N* = 88 (Study 2); Between-subjects	Driving decisions	Parental care motivation (activated vs. control); decision time (fast vs. slow); pedestrian age (child vs. adult)	Utilitarian behavior, preferring to minimize total harm, with saving younger over old. Parental care motivation reduced the probability of hitting a child, especially under time pressure.
Salagean et al. (2024)	*N* = 17; Within-subjects	Self-driving cars	Avatar personalization (personalized vs. generic); motor control (active vs. passive); dilemma congruency (incongruent = harmful action leads to better outcome vs. congruent = harmful action leads to trivial outcome)	Utilitarian behavior, preferring to minimize total harm. Increased arousal for personalized avatars and active motor control. Slower reaction time in active motor control.
Jang and Wallraven (2024)	*N* = 20; Within-subjects	Driving decisions	Pedestrian features (number, age); decision time.	Preference to save children over elderly; lower psychopathy and higher utilitarianism associated with self-sacrifice; high realism reported

### 3.1 VR studies on classic moral dilemmas

The first work found in literature on the study of moral dilemma behavior with VR was authored by Pan and Slater (2011), and consisted of a conference paper presenting results from a small-sample pilot study. Pan and Slater’s (2011) employed a VR scenario that was made to mimic the trolley dilemma, but adopting a different context compared to the original dilemma (e.g., *In an art gallery, an attacker is firing at 5 visitors. Bringing the lift down would save those 5 but put the 1 on the ground floor in danger*). Furthermore, they employed immersive (projected in a room) and non-immersive stimuli (a desktop screen) to present subjects with the dilemma. VR data were then compared to additional online survey data, specifically collected to be used as baseline. Authors found overall comparable results across VR and survey data, however finding a higher prevalence of utilitarian choices both in the VR session and in the post-experimental surveys for subjects in the VR condition. Nevertheless, the study was under-powered, comprising only 36 participants for the VR study and 80 participants for the online survey, and employed a complex mechanism to compare survey data and participants’ behavior.

Compared to Pan and Slater (2011), following two studies (Navarrete et al., 2012; Patil et al., 2014) provided a more precise application of the VR to the trolley dilemma, as they both directly employed a virtual reality representation of the classic dilemma. Both studies were intended to address the judgment-behavior gap, that is, actual behavior may differ from stated judgments (utilitarian vs. deontological) when participants are confronted with a more realistic situation. Specifically, in Navarrete et al. (2012), participants were exposed to a 3D version of the trolley problem in a virtual environment, in a classic between-subject design with subjects randomly assigned to either action condition (i.e., pulling a lever to choose the utilitarian outcome and saving 5 people) or omission condition (i.e., abstaining to pull a lever to choose the utilitarian outcome and saving 5 people). Authors found overall comparable results to previous large-scale surveys, with 90.5% of participants in the action condition choosing the utilitarian outcome, compared to 88.5% in the omission condition. Such difference, however, was not significantly influenced by the experimental condition, although levels of arousal significantly differed, with higher levels of arousal in the action condition. Higher arousal levels were also associated with decreased utilitarian choices, consistently with Greene et al.’s (2001) and Greene’s (2007) dual process model.

Conversely, Patil et al. (2014) employed a within-subjects design, in which participants were requested to complete both text-based versions and VR-versions of the same moral dilemmas, allowing for a more direct comparison of results. Authors found a higher prevalence of utilitarian choices in the VR sessions, with an average proportion of 0.95 (SD = 0.14), compared to the text sessions, in which the average proportion was 0.76 (SD = 0.32). Interestingly, higher discrepancy between judgments and behaviors was found in subjects who were asked to complete the text version first and then the VR version, highlighting the possible role of order effects. In fact, some participants who had previously judged sacrificing one to save many as morally inappropriate in the text version, thus endorsing a deontological approach, switched to a more utilitarian principle “when full spectrum of contextual cues was provided using VR environment” (p. 25). Participants who instead faced the VR version first and then the text-based version showed a stronger tendency to keep consistency in choices across tasks. Finally, contrary to Navarrete et al. (2012), authors found opposite results in terms of emotional arousal: higher arousal was associated with more utilitarian choices. Such results were not consonant with Greene et al.’s (2001) and Greene’s (2007) dual process model, but were explained by the means of another theory, and specifically Cushman’s (2013) adaptation of the same model. This version postulates that the prevalent process behind the judgment depends on whether the focus is on the value assigned to the action or on the value assigned to the outcome. Specifically, both processes have affective content, but for deontological choices, the focus is on the value of the actions, while for utilitarian choices, the focus is on the value of the outcomes. In the VR sessions, participants were more sensitive to outcomes, probably because they could envision the consequences of their choices.

A further development of VR studies on moral dilemmas was Skulmowski et al. (2014), which builds on Navarrete et al. (2012), but implementing several novelties, and specifically: participants were not bystanders of the trolley dilemma, but they were driving the train; they introduced the use of the eye tracker, collecting eye position and pupil dilation; finally, they used repeated measures of the dilemmas. Experimental conditions included the manipulation of several avatar properties, including gender, ethnicity and body orientation. The largest effect was detected in the group dilemma, where authors found a 96% rate of utilitarian decisions across the repeated trials, with participants deciding to sacrifice a single person in order to save the group. Avatars’ gender seemed to play a role, as males were more likely to be sacrificed than females, while body orientation of the avatars did not seem to have a significant effect on participants’ choices. From the analysis of eye movements, in all four conditions, they found an increase in pupil diameter after the moment of decision, suggesting a participants’ increased arousal or cognitive load at that time. Additionally, even in all four conditions, there was a tendency to spend more time looking at the avatar that will be killed in order to reassure themselves of making a “right” decision.

One of the criticisms of presenting the *trolley problem* in VR is that a description of the situation—written or oral—is provided before or during the simulation, leading to a mental simulation that anticipates the decision-making process. To test this, Richesin et al. (2022) conducted a study in which 119 participants were divided into two groups: informed (*N* = 60) and uninformed (*N* = 59). They also recorded psychophysiological measurements, as skin conductance (SC) and heart rate (HR). The results showed that 81.5% of participants across both groups chose the utilitarian action, so there is no significant association between group and choice. Additionally, the decrease of contextual information seems to have no effect on physiological arousal. So, these findings seem to support the dual-process approach proposed by Greene et al. (2001) and Greene (2007) and two previous studies examining trolley problems in VR (Navarrete et al., 2012; Pan and Slater, 2011): the lack of emotional arousal due to the impersonal nature of the classic trolley problem did not call the automatic emotional system into action, leading to the rational utilitarian response. In summary, this study did not find evidence for a significant difference in participants’ decision-making in VR compared to other assessment methodologies, probably due to the impersonal nature of the tested dilemma.

Building on early studies that employed VR to investigate moral actions in *impersonal moral dilemmas*, Francis et al. (2016) was the first to conduct a study using VR to examine decision-making in a *personal moral dilemma*—specifically, the footbridge dilemma, in which individuals are confronted with a high level of emotional conflict. The primary aim was to determine which of the two prevailing theoretical frameworks—Greene’s dual-process theory or Cushman’s action-based model—better accounted for the observed results. The study involved the recruitment of 40 participants who were assigned to two conditions (*N* = 20 each): “action condition” and “judgment condition.” In the first case, participants experienced an audio-visual VR version of the footbridge dilemma in first person view and, using a joystick, they had 10 s to decide whether to push the large person off the bridge to stop an approaching trolley or to let it continue, killing the five people standing on the tracks. After the trolley collided with the large person or with the people on the tracks, participants remained in the virtual environment for 5 s in order to see and understand the consequences of their actions. Instead in the second case, participants read a vignette describing the footbridge dilemma, along with nine distractor dilemmas presented in a random order. They then answered two questions: *“Is it morally acceptable to [specific action]?”* and *“Would you do it?,”* responding with “Yes” or “No” within 10 s.

The results have shown that in the judgment condition, when asked if the action was morally acceptable, 20% of participants endorsed a utilitarian response (i.e., judge that they regard pushing the man as morally acceptable), but only 10% reported they would have actually done it. In the action condition, 70% of participants endorsed a utilitarian response, significantly more than in the judgment condition and the odds of participants endorsing a utilitarian response were 9.33 times higher in the action condition than in the judgment condition. In addition, heart rate change was highest in the action condition. A subsequent study, methodologically identical to the first but conducted with a larger sample, successfully replicated the initial findings. In summary, these results suggest that the visual saliency in VR emphasizes the negative outcome in the moral dilemma and this begins to outweigh the negative value assigned to the action of pushing, in line with Cushman’s theory.

Although the footbridge dilemma in VR is a very “close and personal” paradigm, Francis et al. (2017) decided to investigate even whether and how *haptic feedback* could influence simulated personal moral actions in two studies with VR. So, in order to investigate the relationship between moral judgment and moral action, in the first study they used a robotic haptic interface (vBOT system) to simulate performing a realistic physical action in response to moral dilemmas involving personal force; instead, in the second study there was an interactive sculpture mechanism designed to generate haptic feedback and the sensation of pushing the person off the footbridge.

Even in this case in both studies, the results of moral judgments compared to moral actions were conflicting, as most participants tended to perform utilitarian actions when faced with a *personal dilemma*. Specifically, in the first study, using the vBOT system, participants CHILD could not see the victims but they still provided utilitarian responses. So, these results can be explained as a combination of contextual salience and the different *frame of reference* between judgment and action: the former is based on allocentric considerations and influenced by cultural norms, while the latter is driven by an egocentric perspective.

In a subsequent study, Francis et al. (2019) extended previous virtual research by examining the acute effects of alcohol on moral decision-making, affective empathy, and physiological arousal in both traditional text-based vignettes and VR moral dilemmas. Participants were randomly assigned to placebo, low-alcohol, or high-alcohol conditions and completed moral judgment tasks and virtual action tasks based on the footbridge scenario, alongside behavioral measures of affective empathy. Results revealed a persistent moral inconsistency: participants showed *greater utilitarian actions* in the virtual footbridge dilemma but *fewer utilitarian judgments* in the text-based version, regardless of alcohol condition. These findings corroborate previous evidence of a “saying–doing” discrepancy in moral behavior (Patil et al., 2014; Francis et al., 2016, 2017). Importantly, although alcohol consumption altered affective empathy responses, particularly producing “inappropriate” emotional reactions to facial expressions similar to those found in individuals high in psychopathy, it did not significantly influence either moral judgments or moral actions. These results suggest that the relationship between alcohol, empathy, and moral behavior is more complex than previously assumed and cannot be fully explained by traditional dual-process models or by social-processing accounts alone.

In order to better understand whether different processes guide moral judgments and moral actions, similarly to Francis et al. (2016), Terbeck et al. (2021) tested the footbridge dilemma comparing moral reasons for participants’ choices in the text-based condition vs. VR condition, finding significant differences. Specifically, participants in the VR condition were more prone to refer to the people involved in the dilemma (e.g., they would report to be thinking about the consequences for the people involved) than participants in the text-based condition, who would instead justify their decisions referring to the consequences for themselves. Such results highlight again the different processes from which judgments and behaviors arise (Tassy et al., 2013) and possibly underline that contextual factors in VR sessions (e.g., the possibility to see the victims) induce saliency which ultimately affects individual’s decisions (Patil et al., 2014).

Building on these findings, Maćkiewicz et al. (2023) tried to further explore the explanations behind the utilitarian tendency observed in VR settings by introducing several modifications to the experimental design originally employed by Francis et al. (2016). Changes were applied both to the VR environment and to the textual vignettes used in the questionnaire version. The authors tested two main hypotheses. The first posited that, in Francis et al. (2016), the VR presentation—due to its visual and non-visual salience—may have highlighted the harmful consequences of inaction, thus making outcome-based evaluation prevail over the negative value attributed to the action itself. To test this, they manipulated the visual salience of the negative outcome, predicting an increase in deontological responses as the salience of inaction decreased. The second hypothesis concerned the possibility of diminishing the negative value associated with the action by changing the type of movement required to sacrifice the person standing on the bridge (pushing vs. gesture). According to Cushman’s (2013) theory, this manipulation should produce a stronger utilitarian tendency in the “gesture” condition. The results revealed that in the text-based condition, only a small minority of participants declared that they would sacrifice one person to save five (pushing: 18.18%; gesture: 27.27%). Conversely, almost half of the participants in the VR condition made this utilitarian choice and pushed the woman from the bridge to save the workers (pushing: 52.63%; gesture: 55.26%). Overall, these results did not support the dual-process explanation of the “utilitarian tendency” observed in previous VR studies. Rather, they aligned with the findings of Francis et al. (2016), indicating that the VR effect in the footbridge dilemma is robust across methodological variations. In Study 2, the authors intentionally enhanced the visual and auditory salience of the harmful consequences of inaction but did not observe any significant shift in participants’ responses. This provides further evidence against the proposed dual-process explanation of the utilitarian pattern in VR. Finally, two exploratory meta-analyses reinforced the robustness of the effect and situated these findings within a broader empirical framework of moral decision-making in virtual environments.

Besides the trolley and the footbridge dilemmas, another classic moral dilemma tested in VR include the *Mad Bomber* dilemma. This dilemma deals with the question of whether it is ethical to torture a terrorist in order to save other human beings in an interrogation which has the aim to uncover the location of bombs that will soon self-detonate. This dilemma was tested, along with other 5 trolley dilemma scenarios, by Niforatos et al. (2020), comparing VR sessions with text-based answers.^2^ In their study, they found that participants exhibited a starkly vindictive behavior in the VR when the terrorist was described as plotting another attack. In fact, they detected in VR condition a large increase in adoption of physical torture (+69.6%), and a decrease in psychological tactics (−24.3%).

Another study which used classic moral dilemmas, but with a remote VR technology is Kissel et al. (2023). Authors argue that research on moral dilemmas with VR may still be limited by logistics, as virtually all previous studies have taken place in a laboratory environment, possibly affecting their validity due to observer effects and limiting sample size. Thus, Kissel et al. (2023) tested two versions of the classic trolley and footbridge dilemmas by using commercially available in-home VR headsets, through a third-party website, allowing authors to conduct a “VR-at-a-distance” research. They found overall comparable results with respect to previous lab-based VR studies, with most participants endorsing a utilitarian choice both in the trolley (100%) and in the footbridge (60%) dilemma. Thus, these results suggest that VR-at-a-distance could replicate lab-based experiments.

Finally, the most recent contribution on VR studies on classic moral dilemmas is Liu et al. (2025), which investigated the role of cognitive load and empathy for pain on participants’ choices. Cognitive load refers to the amount of cognitive resources required for processing information, solving problems, and making decisions (Barrouillet et al., 2007), while empathy for pain refers to the perception, judgment, and emotional response to someone else’s pain (Ren et al., 2021). Consistently with previous literature (e.g., Patil et al., 2014; Francis et al., 2016), authors found that under VR conditions, individuals are more prone to utilitarian decisions in impersonal dilemmas (i.e., trolley dilemma), while they are less prone to utilitarian decisions in personal dilemmas (i.e., footbridge dilemma). They also found that individuals under high cognitive load tend to make fewer utilitarian choices, thus intuitively resorting to established moral norms. Finally, in impersonal dilemmas and under VR conditions, individuals with higher empathy for pain tended to make more utilitarian choices, possibly due to the fact that empathy may motivate individuals to reduce overall suffering through increased perception of others’ pain.

### 3.2 VR studies on applied moral dilemmas

VR has opened the door for deeply immersive applications and it allows users to deeply engage in virtual worlds with a sense of presence and embodiment, resulting in increased perceived realism and emotional expression. Thus, in academic research, there are many fields in which VR could be useful to better know how people reason and how they decide to act in a particular scenario (Yin and Xiao, 2024). In this section, we will review works on applied moral dilemmas, including studies focusing on driving decisions of humans and/or of self-driving vehicles, but also studies focusing on other contexts, such as practices in helping professions.

#### 3.2.1 VR studies on driving decisions made by human drivers

In recent years, research on moral reasoning and moral action has not only found a valuable method of assessment and observation through the use of VR but has also identified specific fields of application that are gaining increasing recognition and attention. Driving decisions, for example, represent one of these fields, as they involve situations where individuals are required to make difficult moral choices within a very limited timeframe.

For instance, Bergmann et al. (2018) conducted an extensive VR-based experimental study to explore how participants make moral decisions in traffic scenarios, with the aim of informing the ethical design of self-driving cars. In a series of immersive simulations, participants were asked to choose between ethically challenging options, such as sacrificing themselves to save multiple pedestrians, or choosing between individuals of different ages (e.g., a child vs. an elderly person) or in different situations (e.g., a kneeling man). Across conditions, participants showed a marked tendency toward utilitarian reasoning, preferring to minimize total harm. Results also highlight the importance of contextual variables like age, since participants strongly prioritized saving children over adults or the elderly, but also the kneeling position, which may signal an individual’s need for protection.

Similar core moral preferences were found by Sütfeld et al. (2019), which focused on the methodological aspect of VR studies, comparing the effect of assessment methodologies (text-based vs. VR) on moral decisions in traffic dilemmas. Across two studies, they manipulated the level of abstraction (text-based vs. naturalistic), presentation modality (desktop vs. VR), and response time (slow vs. fast), analyzing how these factors affected the evaluation of victims’ characteristics (e.g., age, gender) and participants’ decisions. They found that core moral preferences remained largely stable across methods, with participants consistently favoring younger over older individuals and females over males. From a methodological point of view, however, differences between text and VR presentations were minimal. Notably, immersive VR did not drastically alter moral choices compared to text-based scenarios, supporting the generalizability of findings from traditional surveys (e.g., Awad et al., 2018) to real-time decision-making in more naturalistic settings.

On a different note, Sütfeld et al. (2017) aims to model human moral-decision making to train models of AI. Authors employed immersive VR to investigate human ethical decision-making in unavoidable collision scenarios relevant to autonomous vehicles. Participants were placed in the driver’s seat of a virtual car and had to choose which of two obstacles—ranging from inanimate objects to animals and humans—they would sacrifice. The authors tested whether human behavior in these moral dilemmas could be predicted by value-of-life-based models (i.e., decision-making models that assume each possible obstacle—such as a person, animal, or object—has a numerical value representing the “worth” or ethical significance of its life). Their findings showed that simple one-dimensional models, such as assigning a fixed value to each obstacle, could successfully capture participants’ decisions, particularly when enough time was available for deliberation. This supports their potential use in the ethical algorithms of self-driving cars, especially in scenarios involving unavoidable collisions. Moreover, the observed decrease in decision consistency under time pressure reinforces the argument for delegating such decisions to machines rather than humans in real-world settings.

On a similar line, Faulhaber et al. (2019) conducted a comprehensive VR study to explore how humans make moral decisions in simulated traffic dilemmas involving self-driving vehicles, focusing on utilitarian decision-making, the influence of contextual cues (victim age, sidewalks), and self-sacrifice. Results consistently showed that participants acted in a utilitarian manner: they prioritized saving the greatest number of avatars and spared younger over older individuals, even when this required driving onto sidewalks or sacrificing themselves. Willingness to commit virtual self-sacrifice emerged when five or more lives could be saved, aligning with findings from Bergmann et al. (2018) and Frison et al. (2016), where many participants were willing to accept self-endangerment under high moral stakes. Moreover, as in Sütfeld et al. (2017), Faulhaber and colleagues argued that moral preferences could be modeled through simple rules based on value-of-life principles (e.g., Disability-Adjusted Life Years).

Another study echoing previous results was Wang et al. (2023), which investigated moral decision-making in a VR traffic dilemma: whether to sacrifice oneself or a pedestrian in the face of unavoidable harm on a mountainous highway. Authors manipulated pedestrian age (child, middle-aged, elderly), pedestrian gender, participant gender, and time pressure (5 s vs. 30s) to study determinants of moral decisions. Also in this case, findings reveal that participants more often chose self-sacrifice, particularly when the pedestrian was a child or a female. Deontological choices were also more common in female participants, who exhibited significantly more self-sacrificial behavior than males. Overall, time pressure amplified self-sacrificing choices.

Continuing to explore the validity of Greene et al.’s (2001) and Greene’s (2007) dual-process theory and the fundamental-motives framework, Zhou and Zhu (2023) investigated whether parental care motivation could influence and affect the type of decision made in a moral dilemma, such as the trolley dilemma, presented in the context of driving scenarios under different time constraints. Additionally, this study aimed to investigate the discrepancy between moral judgments and moral actions in trolley problem decision-making in two studies: one with text-based settings and the other with a VR environment. In the text-based study (Study 1) there were two groups: parental group, in which they used a paradigm to activate parental care motivation, or control group. All participants were presented with two trolley dilemma scenarios. In the one-to-one scenario, one adult and one child were in either lane; in the one-to-many scenario, two adults and one child were in either lane. Participants have to imagine themselves as the driver in these two scenarios and choose which of the two sides to hit. The results showed that people made utilitarian choices. Specifically, participants were inclined to save the child target in the one-to-one scenario, and in addition, parental care motivation had a significant effect only in this scenario. In the VR environment (Study 2), authors divided participants in two groups (parental and control) and they used a novel paradigm to activate parental care motivation in VR to see if parental care motivation would reduce the probability of hitting the child target, predicting this effect would be more substantial when making fast decisions. The results showed that the interaction between parental care motivation and time constraints was significant: the parental group had a significantly lower child hit rate than the control group when making fast decisions. However, when making slow decisions, there was no significant difference in the child hit rate between the two groups. At last, the main effect of parental care motivation was not significant. Overall, the results suggest that parental care motivation can influence the intuitive process of moral decision-making, leading to different choices than those predicted by the dual-process model. Furthermore, the VR environment affects how people process moral dilemmas, promoting more utilitarian decisions when exposed to more immersive visual and auditory details.

Another study that analyzed moral decision-making in time constraints is the one by Samuel et al. (2020). Specifically, they examined whether drivers make utilitarian decisions in situations of unavoidable harm and were forced to make a choice in just 2 s between striking five pedestrians versus one pedestrian (Scenario 1) or no pedestrians (Scenario 2). The results showed that in Scenario 1, only 43% of the participants chose the utilitarian outcome. This proportion is significantly less than Navarrete et al. (2012), which reported a rate of 89% in a 4-s-decision scenario. In Scenario 2, despite the option to maneuver past the five pedestrians altogether, only 62.5% of the drivers successfully evaded the group of five pedestrians. And, even in this case, this proportion was significantly less than that of Navarrete et al. (2012). Thus, the time given to the participants probably plays a fundamental role to obtain ecological information about moral decision-making in a car simulator.

On a different note, Jang and Wallraven (2024) present a novel, multi-stage VR paradigm for studying moral decision-making under time pressure in a realistic emergency scenario. Participants, escaping an incoming tsunami, face sequential dilemmas requiring them to choose whom to save (e.g., child vs. elderly) and ultimately whether to self-sacrifice. Thus, while constituting a VR-based driving simulation study, the main focus is put on time pressure and on the state of emergency. Pilot results confirm prior findings from studies like Faulhaber et al. (2019) and Bergmann et al. (2018), showing a preference for saving children and a willingness to self-sacrifice in high-stakes contexts. Importantly, this study adds a new layer by linking decisions to personality traits (e.g., psychopathy, utilitarianism), highlighting individual differences in moral decisions. For instance, participants who opted for self-sacrifice tended to score lower in psychopathy and higher in utilitarianism.

#### 3.2.2 VR studies on judgments of self-driving vehicles’ moral decisions

Understanding how humans make trolley decisions provides insights into establishing autonomous driving ethical algorithms (Zhou and Zhu, 2023). In fact, these new technologies are requested to act as decision-makers in situations which require choosing morally (e.g., Bonnefon et al., 2016). For instance, in certain situations involving self-driving cars, collision is unavoidable and the machine has to make a decision about which obstacle to collide with in a matter of milliseconds (Sütfeld et al., 2017), possibly balancing the wellbeing of different people, such as pedestrians and passengers. By gathering data on how people make moral judgments in moral dilemmas research, AI engineers can be more informed on how to design autonomous machines that people will use (Awad et al., 2018). As Sütfeld et al. (2017, p. 4) put it, “any implementation of an ethical decision-making system for a specific context should be based on human decisions made in the same context.” From this point of view, research employing VR environments to study moral judgments and behaviors is extremely helpful, as it possibly increases the degree to which engineers can rely on these data, by enhancing their ecological validity (Kissel et al., 2023).

Several studies have investigated the role of contextual variables in influencing moral driving decisions, especially when it comes to self-sacrificing. For example, in a conference paper, Frison et al. (2016) investigated how knowledge about the accident risk influences drivers’ decisions. Specifically, participants had to decide if they wanted their vehicle to swerve and kill others (option A) or stay on track and harm themselves (option B). In option A they manipulated the number, age and personality of pedestrians affected; in option B the outcome for the driver by presenting the probability of their survival. The results showed that the own survival instinct does affect subjects in decision making: with increasing probability of survival, drivers tend to risk their own life to save others and this effect is already significant at a 25% probability of survival. Even when drivers knew they would die in the accident (probability of survival of 0%), they refused to kill others in slightly more than half of the scenarios. Additionally, if more than one person is affected by the accident, subjects decide to risk their own life; if children are injured or even killed, only 18.8% of participants would save their own life; instead, surprisingly, if the pedestrian is a beloved person, this does not significantly affect people’s decisions. So, it emerges that people generally overestimate the control they have over certain situations and believe that the worst cannot happen to them. Furthermore, they seem to prefer their vehicle to make utilitarian choices and risk their life to save others probably because surviving while being aware of being responsible for someone else’s death is more difficult to bear.

De Melo et al. (2021) investigated how people would program autonomous vehicles to act in moral dilemmas involving risk of injury. Across four experiments, participants were asked to decide whether their autonomous vehicles should swerve into a wall (risking the driver) or continue forward (risking five pedestrians), with varying probabilities of injury. Results showed that participants were more likely to choose the utilitarian option—sacrificing the driver to save the pedestrians—when risk to the driver was low and/or risk to pedestrians was high. Importantly, the authors demonstrated that participants’ decisions followed a linear decision function, with switching thresholds that reflected sensitivity to both risk dimensions. In a final experiment, they introduced social influence by presenting participants with normative information about other drivers’ choices. This significantly affected behavior: participants were more likely to choose the utilitarian option when believing others did the same.

On a similar note, Benvegnù et al. (2021) focused on the differences between moral actions in VR-simulated accidents where participants are the drivers and similar situations in which the participants are instead the passengers of self-driving cars. Authors found that participants’ experience when facing moral dilemmas as drivers was overall more negative compared to when they faced it as passengers, showing a greater arousal, a more negative valence, and an increased sense of responsibility in the driving condition. On the other hand, in normal driving conditions, where no killing was involved, participants rated the experience of passengers of self-driving cars as unpleasant. These results suggest that people appreciate control over driving only in peaceful situations, but when their actions may have dire consequences on others, their experience as actual drivers is not preferred over being passengers in autonomous vehicles. Authors argued that although the “contextual richness” of VR enhanced emotional reactions of participants who could vividly experience the situation, it is the need to actively react to the moral dilemmas which most affected the participant’s emotional response.

Also Salagean et al. (2024) used a driving vs. non-driving manipulation, comparing choices and physiological reactions to two accident situations: incongruent dilemmas (i.e., harmful action leading to better overall outcomes, such killing a pedestrian to save themselves and others) and congruent (i.e., refraining from harm when it brought no benefit). Moreover, authors manipulated personalization of the avatar: a subset of the sample, in fact, had been assigned to participate in the simulation using an avatar designed to look like them. While participants showed overall utilitarian tendencies—opting more often to harm one to save many in incongruent trials—personalization and motor control did not significantly affect the number of harmful actions. However, these features did increase physiological arousal (as measured by skin conductance and heart rate), reaction time, embodiment, and presence, suggesting deeper emotional engagement and a heightened sense of agency.

#### 3.2.3 VR studies on other applied moral dilemmas

Use of VR in social science is increasingly becoming more popular even beyond the field of studies focusing on driving decisions. For instance, VR studies may provide insightful results to inform training programs dedicated to specific categories of professionals who often face moral dilemmas.

In this context, Francis et al. (2018) examined whether professionals in the helping professions—such as paramedics and fire service commanders—differ from lay people in how they make and act on moral decisions, particularly in high-stakes situations. Using both text-based and immersive VR versions of the footbridge dilemma, the study explored whether professional training influences not just moral choices, but also emotional and physiological responses during morally challenging situations. The findings replicate prior results (e.g., Francis et al., 2016; Patil et al., 2014) showing that utilitarian decisions are more likely in VR than in text, across all groups. Interestingly, however, professional training did not alter the rate of utilitarian decisions, either in action or judgment, or moral consistency, supporting the idea that classic moral dilemmas may tap into fundamental and training-resistant moral intuitions. On the other hand, trained professionals *did* differ in physiological and emotional responses: fire service commanders and paramedics exhibited lower heart rate reactivity during moral action, and showed significantly less regret following a utilitarian choice, compared to untrained controls. This study extends virtual morality research into occupationally relevant contexts, highlighting the practical utility of VR paradigms for assessing high-stakes moral behavior in populations routinely exposed to moral dilemmas, but also for structuring training programs in ethical decision-making to foster preparedness, emotional calibration, and professional ethics.

### 3.3 Methodological discussions on the use of VR

Besides reporting the empirical results, several of the papers we reviewed briefly discussed the importance and sometimes the limitations of using VR in studies on moral dilemmas. In fact, especially the first papers in this new branch of literature (e.g., Navarrete et al., 2012; Patil et al., 2014) had a methodological nature: they aimed not only to test psychological theories of moral behavior and judgments, but they also were intended to validate the use of a new technology as an assessment method. As a consequence, in the introduction and discussion sections of their papers, authors would often discuss the reasons why they adopted this new methodology, its advantages and limitations. This section reviews these methodological discussions, and then it focuses on the work of methodologists (e.g., Ramirez, 2019; Rueda and Lara, 2020) which critically assesses the epistemological status of this new approach. Throughout the papers, we were able to identify 6 macro-arguments that scholars used to justify and explain the use of VR in moral dilemmas (summarized in Table 3).^3^

**Table 3:** The 6 macro-arguments that scholars used to justify and explain the use of VR in moral dilemmas in the papers we reviewed.

Reasons to adopt VR	Explanation	Sources
Enhancing ecological validity	VR allows for an immersive and fully interactive experience of social situations, making VR data a better approximation of real life behaviors while maintaining experimental control (Parsons et al., 2017)	Pan and Slater (2011) Patil et al. (2014) Skulmowski et al. (2014) Frison et al. (2016) Francis et al. (2016) Francis et al. (2018) Bergmann et al. (2018) Faulhaber et al. (2019) Niforatos et al. (2020) Samuel et al. (2020) Terbeck et al. (2021) Benvegnù et al. (2021) De Melo et al. (2021) Richesin et al. (2022) Kissel et al. (2023) Zhou and Zhu (2023) Wang et al. (2023) Salagean et al. (2024) Yin and Xiao (2024) Liu et al. (2025)
Addressing judgment-behavior gap	Actual behavior may differ from stated judgments (utilitarian vs. deontological) in a more realistic situation. As such, VR may be a better tool to investigate actual behavior	Pan and Slater (2011) Navarrete et al. (2012) Patil et al. (2014) Francis et al. (2016) Francis et al. (2017) Francis et al. (2018) Faulhaber et al. (2019) Terbeck et al. (2021) Richesin et al. (2022) Kissel et al. (2023) Salagean et al. (2024) Jang and Wallraven (2024)
Conducting ethically unacceptable experiments	VR allows researchers to build designs which would be considered as ethically unacceptable if they were reproduced in real life as they would be too risky (e.g., car accidents) or that would require a high degree of experimental deception (e.g., Milgram’s experiments)	Pan and Slater (2011) Navarrete et al. (2012) Skulmowski et al. (2014) Patil et al. (2014) Niforatos et al. (2020) Benvegnù et al. (2021)
Possibility to run manipulations in a controlled environment	VR simulates moral conflicts in real-life scenarios with a high degree of customizability of the environment and of the contextual cues, allowing numerous manipulations	Patil et al. (2014) Sütfeld et al. (2017) Bergmann et al. (2018) Faulhaber et al. (2019) Niforatos et al. (2020) Benvegnù et al. (2021) De Melo et al. (2021) Richesin et al. (2022) Liu et al. (2025)
Time constraints	VR is able to incorporate into decision-making a “time frame long enough to allow responses that are not time-pressured but short enough to prevent a long elaborate decision-making process that would be unrealistic in an action framework” (Francis et al., 2017, p. 3)	Patil et al. (2014) Francis et al. (2016) Francis et al. (2017) Sütfeld et al. (2017) Bergmann et al. (2018) Sütfeld et al. (2019) Samuel et al. (2020) Benvegnù et al. (2021) Zhou and Zhu (2023)
Integration of multimodal data	VR allows researchers to simultaneously collect data coming from different sources (i.e., eye movements, skin conductance, heart rate) and in different formats, and integrate them to investigate multiple processes contextually	Navarrete et al. (2012) Patil et al. (2014) Skulmowski et al. (2014) Francis et al. (2018) Richesin et al. (2022) Salagean et al. (2024) Liu et al. (2025)

#### 3.3.1 Enhancing ecological validity

The first main argument, which is mentioned by almost all of the papers we reviewed, is the ability of VR to enhance the ecological validity of studies on moral dilemmas. Text-based scenarios, in fact, cut out many important contextual information which is instead naturally provided by VR, invoking feelings of presence, making participants’ responses more similar to what they would have been like in a real scenario (Kissel et al., 2023). VR, in fact, increases contextual saliency of presentational features of the dilemmas, affecting participants’ choices (Patil et al., 2014). Compared to text-based scenarios, virtual environments are more able to engage with emotional processing, and that is reflected in skin conductance data which show that VR moral dilemmas are more emotionally arousing (e.g., Patil et al., 2014; Skulmowski et al., 2014). In this context, as Yin and Xiao (2024) note, the meaningfulness of the experience is a key driver of ecological validity, as it refers to the degree to which participants feel as though their virtual actions have real consequences. First-person VR applications, they argue, may appear as more immersive and generate “increased sensations of body representation, stronger emotional responses, and heightened feelings of presence when compared to the third-person” (p. 394:2). However, as Navarrete et al. (2012, p. 365) note, this does not mean that scholars in this research program claim “that the actions conducted within VR are identical to those committed in the grounded world outside of the research lab, given that actions in the latter clearly have greater legal, reputational, physical, and long-term emotional consequences for the actors.” The main contribution of virtual environments to studies on moral dilemmas is providing an “important intermediate step” that allows researchers to collect data which may be a better approximation of real-world behaviors than textual answers to hypothetical scenarios.

#### 3.3.2 Addressing judgment-behavior gap

Text-based scenarios are often criticized due to the fact that they measure moral judgments, rather than actual moral behaviors (e.g., Patil et al., 2014). The problem is that actual behaviors may plausibly differ when people face actual moral dilemmas—the classic problem of not “practicing what you preach” (Francis et al., 2016). In this regard, several studies have shown conflicting results and highlighted the presence of a gap between moral judgment and moral action. For instance, when making a moral decision in a personal moral dilemma such as *the footbridge dilemma*, most people’s judgments were based on deontological reasoning, whereas their actual moral actions were guided by utilitarian considerations (Francis et al., 2016). While this may appear as a purely theoretical problem when it comes to classic and totally hypothetical moral dilemmas (e.g., trolley problem or footbridge dilemma), it has real-world relevance when it comes to driving decisions. While unfortunate, tragic driving scenarios in which people and/or autonomous vehicles may face moral dilemmas are plausible, thus data on how decision-makers would actually act are both useful to build preparedness in human drivers and to design ethical algorithms to instruct autonomous vehicles in such problematic instances. In this sense, virtual environments are used “as a small step in forging a link between moral judgment and moral behavior involving otherwise intractable behavioral content (i.e., killing for the greater good), as it occurs in real time” (Navarrete et al., 2012, p. 365). Therefore, the use of innovative tools such as virtual reality could represent a crucial step in gaining a deeper understanding of the dynamics between moral judgment and moral action, as well as in developing ethical strategies applicable to real-world contexts, such as autonomous driving (Salagean et al., 2024).

#### 3.3.3 Conducting ethically unacceptable experiments

The simulated nature of virtual environments allow experimenters to conduct research which would be unethical to conduct in real life. For instance, scenarios that involve extreme harm to others could not obviously be accurately reproduced in real life (Navarrete et al., 2012). This includes all situations described in classic moral dilemmas, which almost always entail some degree of violence, including tragic accidents (e.g., trolley dilemma), killing (e.g., footbridge dilemma) or torture (e.g., mad bomber dilemma), but also driving dilemmas, where pedestrians and/or passengers are at risk of being fatally injured and drivers sometimes decide to self-sacrifice. Traditionally, psychological studies which involved some (supposed) degree of violence had to resort to a high degree of experimental deception. Milgram’s (1963) experiments on obedience to authority provide a good example, as participants would typically believe that they were causing real harm to other participants. However, ethical norms on experimental practices generally condemn the use of experimental deception, especially when violence is involved (Baumrind, 2013). Not for nothing, one of the most frequently cited papers by VR studies on moral dilemmas is the famous VR replication of Milgram’s experiment by Slater et al. (2006), criticized as a virtually real experience “capable of traumatizing its subject despite its virtual nature” (Ramirez, 2019, p. 226).

#### 3.3.4 Possibility to run manipulations in a controlled environment

While text- and graphic-based questionnaires offer a high degree of experimental control, they also present a significant limitation: by stripping dilemmas of their non-essential contextual features, they risk oversimplifying complex moral scenarios and thereby limiting the generalizability of the findings. The result is a limited participant’s engagement with the targeted experimental manipulation (Patil et al., 2014). The use of VR allows for the creation of a more immersive and ecologically valid environment, in which experimental stimuli provide contextual information similar to that available in real-life situations, rather than being restricted to a necessary and sufficient amount of information. Furthermore, VR enables the manipulation of key characteristics of the individuals involved in moral dilemmas, allowing researchers to investigate whether and how certain variables—such as age, gender, or the spatial orientation of the subjects—affect moral decision-making processes. Prior research has demonstrated that children are more frequently prioritized over adults, suggesting that the age of potential victims plays a crucial role in moral decision-making (Sütfeld et al., 2017). In the context of autonomous driving vehicles, certain traffic-specific factors also warrant consideration. For example, sidewalks serve as designated safe spaces for pedestrians, which may lead to an internalized reluctance to drive onto them, thereby influencing decision-making in a modified version of the trolley dilemma. Moreover, there are scenarios in which individuals may only be able to save lives by sacrificing their own, raising further ethical complexities (Faulhaber et al., 2019).

#### 3.3.5 Time constraints

Moral choices are often made in conditions of time pressure, which, according to dual process theories, inevitably influences the psychological mechanisms (intuitive vs. deliberate) involved in the decision-making processes. While it is possible to put time constraints on text-based studies of moral dilemmas, “the natural passing of time is a feature inherent to VR studies” (Sütfeld et al., 2017, p. 3) and it largely contributes to the feeling of immersion and thus to the ecological validity of the results. This is particularly evident in driving dilemmas. For instance, Samuel et al. (2020, p. 2) made drivers decide in a scenario which mimicked the trolley dilemma in just 2 s to keep the experimental duration close “to the time drivers have to make a life or death decision going at freeway speeds in heavy traffic.”

#### 3.3.6 Integration of multimodal data

Virtual environments allow researchers to collect data that go well beyond decision outputs and behaviors, and relate to physiological reactions to the simulated experiences participants go through during the experiment. The contextual richness of VR is much better suited to activate emotional responses of participants compared to the static format of text-based scenarios (e.g., Patil et al., 2014). In this regard, Navarrete et al. (2012), for instance, recorded electrodermal activity to assess autonomic arousal in a VR experiment and they state, “these findings are important, as they affirm the empirical link between emotion and moral action, and provide preliminary evidence that similar neurophysiological processes may mediate moral judgment and action” (p. 368). Furthermore, the use of psychophysiological measures not only establishes an empirical foundation for exploring the contexts in which moral judgment and action may diverge, but also highlights the potential for integrating physiological measures into VR study designs to assess arousal alongside self-reports (Skulmowski et al., 2014; Richesin et al., 2022). Finally, the analysis of multimodal data allows to obtain a more comprehensive understanding of the interplay between cognitive and emotional processes in moral decision-making.

It is worth noting that some methodologists criticized the use of VR simulations on the very basis on which experimenters defended and supported their use. For instance, (Ramirez and LaBarge, 2018, p. 252) criticized “virtually real experience”—i.e., “experiences treated by subjects *as if* they were real experiences, via some combination of behavioral, physiological, neurological, or cognitive similarities between virtual and real experiences.” In their view, virtual simulations are too accurate in depicting the subjective point of view of participants (*perspectival fidelity*) and they appear as too plausible in terms of experience (*context-realism*). From the experimenter’s point of view, the combination of these two features enhances the ecological validity of the results, but it may also raise ethical concerns. In fact for some authors the strength of VR is that “allows us to conduct experiments that would be ethically unacceptable to execute in non-virtual environments” (Skulmowski et al., 2014, p. 2), but others disagree: for instance, one potential risk is that the sense of presence facilitated by VR media may lead to permanent psychological or biological changes in users; additionally, the specific ways in which individuals are embodied in VR can have long-term behavioral effects (Ramirez and LaBarge, 2018).

Despite these concerns, the continued development of VR technology remains highly promising due to its vast potential and significant contributions to research. However, it is essential to advance this technology while adhering to the heuristic principle of “The Equivalence Principle (TEP)” that serves, in part, as a framework for promoting caution and ethical responsibility in the design and implementation of VR environments capable of generating virtually real experiences. As The Equivalence Principle (TEP) suggests: “if it would be wrong to allow subjects to have a certain experience in reality, then it would be wrong to allow subjects to have that experience in a virtually real setting” (Ramirez and LaBarge, 2018, p. 250). This principle reinforces the idea that ethical concerns should not be disregarded simply because an experience takes place in a virtual environment rather than in the physical world.

Another strength of using VR is its potential to enhance the ecological validity of experimental results. Ecological validity is achieved when researchers can confidently assume that the behaviors, reactions, or decisions measured in a VR environment align with those that subjects would exhibit in a more naturalistic context. However, in this regard, Ramirez (2019) argued that several moral dilemma studies conducted in VR environments (Navarrete et al., 2012; Patil et al., 2014; Francis et al., 2016; Sütfeld et al., 2017) lack ecological validity due to specific design elements that reduce both context-realism and perspectival fidelity. Specifically, in the studies conducted by Navarrete et al. (2012), Patil et al. (2014), and Francis et al. (2016), participants were immediately placed into the decision-making simulation without any contextual information explaining their presence in the scenario, nor was such information naturally integrated into the simulation itself. Additionally, participants were not provided with any logical rationale for their presence in the environment. These design choices likely increased the perception of the simulation as artificial, leading subjects to adjust their moral expectations accordingly.

In Patil et al. (2014), for instance, participants were suspended above the train tracks with a ‘god-like’ perspective over the rail yard, further diminishing both the context-realism and perspectival fidelity of the scenario. Similarly, in Francis et al. (2016), the use of a non-diegetic voiceover to inform participants about the presence of the train and the possibility of pushing a man onto the tracks not only reduced perspectival fidelity and context-realism but also externalized the source of the idea to push, distancing it from the subject’s own moral reasoning. In Navarrete et al. (2012), context-realism was further diminished by the fact that the virtual agents standing in the path of the simulated trolley exhibited no signs of rational agency. Additionally, the environment itself was barren, lacking any meaningful features that would make it a believable setting for a moral decision. Meanwhile, in Patil et al. (2014), the study’s iterative structure may have contributed to a diminished sense of realism, as participants were exposed to eight different virtual dilemmas in rapid succession.

Ramirez (2019) further noted that while Patil et al. (2014) depicted the deaths of virtual subjects, these depictions lacked realism for two reasons. First, while virtual characters perished with some degree of bloody accuracy, the remaining virtual agents remained motionless. Second, participants were quickly ushered into subsequent dilemmas regardless of their previous choices, further undermining the perceived realism of the experience and reducing it to something more akin to a video game scenario.

In contrast, in Sütfeld et al. (2017) the study’s context-realism was compromised not only because participants were not given any background on why they found themselves in the vehicle, but also because they controlled the car using a keyboard, which reduced perspectival fidelity. Additionally, the use of an audio tone to signal the need for moral decision-making and indicate the time available for such decisions further detracted from the naturalism of the scenario. Nevertheless, despite these limitations, Sütfeld et al.’s (2017) study also incorporated several design elements that enhanced context-realism and perspectival fidelity. For instance, the scenario was presented from a naturalistic, first-person perspective, which increased immersion. Furthermore, participants remained seated throughout the experiment, reinforcing a sense of embodiment and increasing perspectival fidelity.

But even if researchers were able to overcome the practical problems related to context-realism and perspectival fidelity—which has been defined as insurmountable by Ramirez and LaBarge (2020)—, thus succeeding in creating a virtually real experience, they would still face serious ethical issues. In fact, a virtually real experience of a moral dilemma would most likely expose participants to a traumatic experience, such as pushing a man off a bridge. However, as Ramirez and LaBarge (2020) argue, the biggest problem from an ethical point of view is that dilemmas such as the footbridge would inculcate immoral behaviors or judgments in participants. In fact, the footbridge dilemma leads participants to consider the person who will be pushed off as a mere solution to a problem, with no possibility to ask for their consent. In this sense, the footbridge dilemma violates the so-called Doctrine of the Double Effect (Lotto et al., 2014), according to which it is permissible to cause harm for a greater good as long as it is foreseen and unintended (Aquinas, 1952/1274). Ramirez and LaBarge (2020) discuss that seeing someone else’s bodies as instrumental solutions might be considered arguably immoral by the general population, and that VR experiences of such dilemmas require participants to *be* someone who they would rather not be morally wise. These ethical considerations would make these designs “impermissible to deploy” (p. 3329).

## 4 Discussion

In our study, we found that existing research generally suggests a consistent trend toward more utilitarian choices in VR compared to text-based paradigms (Pan and Slater, 2011; Navarrete et al., 2012; Patil et al., 2014; Skulmowski et al., 2014; Richesin et al., 2022; Kissel et al., 2023). However, the magnitude and interpretation of this effect vary considerably across studies, reflecting both methodological heterogeneity and differences in the emotional and contextual features of the dilemmas employed, underlining the ambivalence of theoretical interpretations.

This field of research essentially builds upon two theoretical frameworks of moral decision-making. First, the dual-process theory proposed by Greene et al. (2001) and Greene (2007) suggests that moral decision-making arises from the interaction between two distinct cognitive systems: an intuitive-emotional system and a deliberative-rational system. The intuitive-emotional system operates quickly and automatically, relying on immediate affective responses and it is typically associated with deontological judgments, which reject morally unacceptable actions regardless of their consequences, particularly in personal dilemmas involving direct harm. In contrast, the deliberative-rational system is slower, controlled, and based on cost–benefit analysis. This system underlies utilitarian decision-making, where maximizing overall well-being takes precedence. So according to this theory, moral dilemmas create a conflict between these two processes: the intuitive-emotional system tends to inhibit actions perceived as inherently wrong, while the deliberative-rational system supports utilitarian choices when analytical reasoning overrides emotional responses. Instead, according to Cushman’s (2013) framework, both deontological and utilitarian decision-making processes have an affective component. The key factor determining which process is activated depends on attentional focus: deontological choices arise when individuals prioritize the moral value of the action itself, whereas utilitarian decisions emerge when attention is directed toward the consequences of the action. In VR sessions, the increased tendency toward utilitarian choices may be explained by participants focusing more on the outcomes of their actions rather than on the moral evaluation of the action itself.

When examining impersonal dilemmas such as the trolley problem, several studies (e.g., Pan and Slater, 2011; Navarrete et al., 2012; Kissel et al., 2023) found that participants tend to act utilitarian in VR, typically sacrificing one to save five. Yet, these studies also differ in how arousal and presence were measured and interpreted. For instance, Navarrete et al. (2012) reported that higher arousal was linked to fewer utilitarian choices, consistent with Greene et al.’s (2001) and Greene’s (2007) dual-process model, Patil et al. (2014) observed the opposite pattern, with heightened arousal correlating with more utilitarian behavior, possibly aligning with Cushman’s (2013) model. Further contrasting results were observed in the study by Richesin et al. (2022), where utilitarian choices in VR were not accompanied by increased psychophysiological arousal, aligning with Greene’s model. Moreover, Skulmowski et al. (2014) did not find any relationship between behaviors and affective state, as “apparently, the utilitarian action tendency is too strong to be interfered by affective responses” (p. 12).

In personal dilemmas, such as the footbridge scenario, immersive and interactive VR consistently amplifies utilitarian responding compared to textual versions (e.g., Francis et al., 2016, 2017, 2019; Terbeck et al., 2021; Maćkiewicz et al., 2023). These studies collectively suggest that first-person perspective, embodiment and sensory realism shift attention from moral norms towards outcome-based reasoning. In line with Cushman’s framework, as the visual saliency may enhance the perception of negative consequences, outweighing the negative valuation of the action itself. Notably, Francis et al. (2017) further demonstrated that introducing haptic feedback reinforced the utilitarian trend, suggesting that sensorimotor engagement plays a role in shaping moral behavior.

Taken together, the reviewed studies reveal convergent evidence that VR amplifies outcome-oriented, utilitarian decision-making, but also underscore substantial inconsistency in emotional arousal, making it difficult to test theoretical predictions and compare interpretations. Future work should therefore aim to systematically compare these variables through consistent paradigms, developing standardized measures and parameters to better integrate findings across studies.

Finally, more recent contributions expand this research toward methodological innovation and contextual diversity. Niforatos et al. (2020) introduced the *Mad Bomber* dilemma—the only study departing from trolley-type scenarios—showing that VR elicits more punitive and physically aggressive tactics, hinting at broader applications in the study of moral conflict. Similarly, Kissel et al. (2023) demonstrated that remote, in-home VR setups can reproduce laboratory findings, addressing prior scalability limitations.

Across applied settings—primarily traffic and driving decisions—VR studies converge on utilitarian trend: frequently decide to minimize overall harm by sacrificing fewer lives, often prioritizing specific victim characteristics such as age or vulnerability (Frison et al., 2016; Bergmann et al., 2018; Faulhaber et al., 2019; Wang et al., 2023). One consistent finding is the preference for saving younger individuals over older ones, with children receiving the highest priority across multiple studies (Bergmann et al., 2018; Sütfeld et al., 2017, 2019; Wang et al., 2023). This pattern aligns with theories of value-of-life prioritization on moral decision-making. Moreover, in some studies, gender also played a role, with female pedestrians being saved more often than male pedestrians (Wang et al., 2023; Sütfeld et al., 2019). Additionally, the willingness to self-sacrifice under certain conditions is frequently observed, particularly when multiple lives can be saved (Frison et al., 2016; Faulhaber et al., 2019; Wang et al., 2023).

A second major theme is the role of contextual and situational factors in shaping moral choices. Studies manipulating variables such as time pressure and decision framing consistently show that these factors significantly influence outcomes. Specifically, different studies (e.g., Sütfeld et al., 2017; Samuel et al., 2020) found that under time pressure, decision-makers fail to make utilitarian decisions, sometimes increasing the likelihood of self-sacrifice (Wang et al., 2023), possibly due to impaired deliberation. Similarly, environmental cues, such as the presence of sidewalks or perceived vulnerability (e.g., a kneeling individual), also modulate participants’ responses (Bergmann et al., 2018; Sütfeld et al., 2019). Agency and roles too play a role, as being a driver vs. passenger increases arousal and perceived responsibility, without necessarily changing the utilitarian direction of choice (Benvegnù et al., 2021). Finally, sacrificial decisions may also depend on the situation’s risk structure: people make less utilitarian choices when self-risk is higher and more when risk to pedestrians is lower (De Melo et al., 2021).

From a methodological perspective, research comparing VR-based moral dilemmas with traditional text-based assessments suggests that while core moral preferences remain stable across modalities, immersive environments enhance emotional engagement and saliency, potentially leading to more ecologically valid decision-making (Sütfeld et al., 2019; Zhou and Zhu, 2023). However, in some cases, VR did not drastically alter moral choices compared to text-based scenarios, suggesting that conventional survey-based findings (e.g., Awad et al., 2018) may still provide relevant insights into real-world moral cognition (Sütfeld et al., 2019).

Another crucial aspect involves the implications for autonomous vehicle ethics and AI modeling. A few studies suggest that human moral decisions in driving scenarios can be approximated using value-of-life-based models, which assign numerical worth to different entities (Sütfeld et al., 2017; Faulhaber et al., 2019). This approach provides a potential framework for designing ethical decision-making algorithms for self-driving cars, reinforcing the idea that machines may be better suited than humans to handle moral dilemmas under high-stakes time constraints (Sütfeld et al., 2017; Samuel et al., 2020).

Finally, individual differences in moral reasoning are beginning to be explored more thoroughly. Jang and Wallraven (2024) introduce a novel multi-stage VR paradigm linking moral decisions to personality traits such as psychopathy and utilitarianism, revealing that those scoring lower in psychopathy and higher in utilitarianism are more likely to opt for self-sacrifice. This suggests that moral thresholds vary across individuals, and future research should further investigate the interaction between personal traits and moral behavior in VR simulations.

In conclusion, this review has some limitations. The absence of a formal systematic review protocol may reduce reproducibility and coverage. Furthermore, the high methodological heterogeneity among the included studies—both in terms of VR implementation and experimental manipulations—may also have affected comparability across results. However, these limitations were mitigated by explicitly reporting search criteria and by consulting additional databases. Acknowledging these aspects underscores the exploratory nature of this work and provides a foundation for more systematic and standardized future reviews.

## 5 Conclusion

VR offers a powerful methodological advantage in the study of moral dilemmas by enabling the simulation of realistic, immersive, and emotionally salient environments that surpass the limitations of abstract, text-based paradigms. This immersive quality enhances ecological validity by capturing actual moral behavior rather than mere verbal judgments, particularly in scenarios such as driving dilemmas or interactions involving autonomous systems, where realistic decision-making data are critical (e.g., Sütfeld et al., 2017; Kissel et al., 2023). Moreover, VR allows for the integration of multimodal data—including physiological and self-report measures—facilitating a more comprehensive analysis of the emotional and cognitive processes involved in moral reasoning (Francis et al., 2017; Richesin et al., 2022; Liu et al., 2025). Importantly, VR also enables the study of moral cognition as it evolves dynamically over time, within temporally structured and contextually rich scenarios, offering insights into how shifting sensory cues and situational pressures modulate moral decisions in real time.

Despite its numerous strengths, we argue that the field of VR research on moral dilemmas suffers from several limitations. A first major concern is that the application domain remains highly concentrated in the area of autonomous vehicles, with the majority of VR studies focusing on trolley-type driving scenarios (e.g., Sütfeld et al., 2017; Faulhaber et al., 2019; Wang et al., 2023). While these paradigms provide a controlled setting to explore sacrificial decisions in a field that is increasingly gaining societal relevance, they risk limiting the scope of moral research. As some scholars have suggested (e.g., Rueda and Lara, 2020), VR holds promise far beyond driving—particularly in fields such as medical ethics, professional training (Francis et al., 2017), and intergroup conflict (Hasler et al., 2021)—yet these applications remain underexplored in experimental psychology of moral dilemmas.

A second limitation concerns the high variability in moral experimental paradigms, which, while enabling flexibility and creativity, also introduces challenges for replicability and comparison across studies. Each VR setup tends to be highly customized in terms of scenario design, visual realism, avatar features, embodiment, and interaction style. This can significantly affect participants’ psychological responses if the VR environment fails to accurately model the situational features of paradigmatic moral judgments (Ramirez, 2019). For instance, the relatively simple, action-focused VR paradigm employed by Navarrete et al. (2012) differs substantially from the richer, real-time interactive simulations used in studies like Richesin et al. (2022), making it difficult to isolate core psychological mechanisms or draw generalizable conclusions about moral cognition. This variability complicates the development and testing of theoretical models—the debate over Greene’s and Cushman’s models provides a good example—and underscores the need for standardized protocols or reporting guidelines.

In addition, in practical terms, VR research presents logistical and technical barriers that can limit its accessibility and scalability. Unlike traditional survey-based studies, VR experiments typically require specialized equipment, physical lab space, and trained personnel to manage both the technological and psychological aspects of the experience. These requirements can limit the scalability and accessibility of VR studies, especially for large or diverse samples. Furthermore, the integration of psychophysiological measures demands additional layers of coordination, often requiring participants to remain tethered to stationary equipment, which in turn can constrain experimental realism and participant movement.

Moreover, methodologically wise, VR studies are not immune to social desirability bias (i.e., a tendency in participants to answer in accordance with social norms instead of their true beliefs). This phenomenon may systematically distort the way people report moral judgments or actions, for instance, inflating the rate of utilitarian answers in the footbridge dilemma. Although different authors acknowledge the possible presence of social desirability bias in their results (e.g., Frison et al., 2016; Sütfeld et al., 2017; Terbeck et al., 2021), only a handful of studies actually controlled for social desirability. For instance, Sütfeld et al. (2019) found marginal evidence for higher scores on a social desirability scale predicting larger omission bias and gender bias towards sacrificing males over females. Also Skulmowski et al. (2014) found similar results, with subjects reporting higher levels of social desirability bias also tending to sacrifice the male avatars instead of female ones.

To date, studies using VR have primarily focused on variations of the trolley dilemma and the footbridge dilemma. Only Niforatos et al. (2020) have presented a study in which the “Mad Bomber” dilemma was tested in VR. In the future, VR could be employed to investigate other dilemmas, such as the “Tennis Tournament” dilemma (Spranca et al., 1991), that explores the conflict between personal gain and ethical responsibility, or “The Witness” dilemma (Spranca et al., 1991) that explores the conflict between personal responsibility and commitment to social justice.

Future research may develop along several new lines. First, while VR is already employed in professional education and training, an important and underexplored direction concerns the adaptation of classic moral dilemmas to professional contexts, such as deciding whom to rescue for firefighters or paramedics (Birtill et al., 2021). These applied versions of moral dilemmas could enhance ecological validity and provide new insights into moral reasoning under real-world constraints, ultimately fostering greater emotional resilience and ethical consistency in professional contexts. Second, longitudinal designs could help determine whether repeated exposure to virtual moral scenarios influences moral sensitivity, emotional regulation, or behavioral consistency over time. Third, future studies could increase ecological realism through greater personalization and real-time social interaction. Only one existing study has implemented avatar personalization (Salagean et al., 2024), but future work may test what happens when victims or agents within the scenario correspond to real, simultaneously participating individuals, or when decision-making is shared among multiple users. Such interactive paradigms could shed light on social phenomena such as diffusion of responsibility and collective moral reasoning. Finally, integration with AI-driven agents could enable adaptive and context-sensitive simulations, allowing researchers to investigate how artificial intentionality and moral responsiveness influence human moral judgment and behavior in dynamic environments.

To achieve broader theoretical and practical impact, researchers should strive for greater methodological standardization across studies. Our review revealed a high degree of methodological heterogeneity among the studies. This variability concerns both the technical implementation of VR and the experimental manipulations adopted. From a technological standpoint, the reviewed studies differ substantially in terms of immersion level, hardware, and interface design—ranging from desktop-based setups to fully immersive head-mounted displays or CAVE systems. Such diversity affects the degree of presence, embodiment, and emotional engagement, making it difficult to directly compare behavioral outcomes across studies. At the same time, researchers have employed different operationalizations of moral dilemmas, often modifying the original paradigms (e.g., Footbridge, Trolley) or introducing unique contextual details and response modes. While these differences enhance understanding of moral reasoning, they complicate the interpretation of utilitarian versus deontological choices, as variations in sensory realism, task framing, and action requirements may all influence moral responding.

Future research would benefit from efforts toward greater standardization, including the development of shared VR protocols, validated stimuli repositories, and transparent reporting of technical parameters. Such measures would enhance replicability and allow more meaningful comparisons across studies. Additionally, innovations such as remote or in-home VR experimentation (Kissel et al., 2023) may offer a promising solution to some of the current limitations of this research field. Conducting VR studies traditionally requires specialized technical expertise, dedicated laboratory spaces, and costly equipment, which restricts both scalability and participant diversity. Remote VR paradigms allow researchers to collect data in participants’ own environments, potentially increasing inclusivity and sample heterogeneity while reducing logistical barriers. Nevertheless, this approach must carefully address issues of data quality, experimental control, and ethical oversight, to ensure that methodological flexibility does not compromise internal validity. Overall, the future of VR-based moral research lies in its ability to combine ecological validity, technological accessibility, and ethical responsibility.

## Footnotes

1 This theory has traditionally been used to explain the differences observed in choices between trolley and footbridge dilemmas. However, it has received both methodological (McGuire et al., 2009) and theoretical (Cushman, 2013) criticism and revisions. This ambiguity is also reflected in the empirical studies we review in this work, where results are sometimes consistent with Greene’s account and at other times align more closely with Cushman’s perspective.

2 Technically speaking, this is a conference paper, but we decided to include it in this review because it introduces a new moral dilemma called “Mad Bomber Dilemma.”

3 This identification of 6 macro-arguments is clearly not exhaustive and it only serves as a way to systematize the main methodological principles mentioned by authors. Thus, we do not exclude that there may be other valid reasons to adopt VR technologies as an assessment methodology. Furthermore, we admit that some of these arguments are interrelated: for instance, the use of VR to address the judgment-behavior gap could be considered as a way itself to enhance ecological validity.
